# Autoantibodies in patients with obsessive-compulsive disorder: a systematic review

**DOI:** 10.1038/s41398-023-02545-9

**Published:** 2023-07-03

**Authors:** Dominik Denzel, Kimon Runge, Bernd Feige, Benjamin Pankratz, Karoline Pitsch, Andrea Schlump, Kathrin Nickel, Ulrich Voderholzer, Ludger Tebartz van Elst, Katharina Domschke, Miriam A. Schiele, Dominique Endres

**Affiliations:** 1 Department of Psychiatry and Psychotherapy, Medical Center - University of Freiburg, Faculty of Medicine, University of Freiburg, Freiburg, Germany; 2 Department of Psychiatry and Psychotherapy, University Hospital, LMU Munich, Munich, Germany; 3 Schoen Clinic Roseneck, Prien am Chiemsee, Germany

**Keywords:** Diagnostic markers, Molecular neuroscience

## Abstract

Obsessive-compulsive disorder (OCD) is a frequent and debilitating mental illness. Although efficacious treatment options are available, treatment resistance rates are high. Emerging evidence suggests that biological components, especially autoimmune processes, may be associated with some cases of OCD and treatment resistance. Therefore, this systematic literature review summarizing all case reports/case series as well as uncontrolled and controlled cross-sectional studies investigating autoantibodies in patients with OCD and obsessive-compulsive symptoms (OCS) was performed. The following search strategy was used to search PubMed: “(OCD OR obsessive-compulsive OR obsessive OR compulsive) AND (antib* OR autoantib* OR auto-antib* OR immunoglob* OR IgG OR IgM OR IgA)”. Nine case reports with autoantibody-associated OCD/OCS were identified: five patients with anti-neuronal autoantibodies (against N-methyl-D-aspartate-receptor [NMDA-R], collapsin response mediator protein [CV2], paraneoplastic antigen Ma2 [Ma2], voltage gated potassium channel complex [VGKC], and “anti-brain” structures) and four with autoantibodies associated with systemic autoimmune diseases (two with Sjögren syndrome, one with neuropsychiatric lupus, and one with anti-phospholipid autoantibodies). Six patients (67%) benefited from immunotherapy. In addition, eleven cross-sectional studies (six with healthy controls, three with neurological/psychiatric patient controls, and two uncontrolled) were identified with inconsistent results, but in six studies an association between autoantibodies and OCD was suggested. In summary, the available case reports suggest an association between OCD and autoantibodies in rare cases, which has been supported by initial cross-sectional studies. However, scientific data is still very limited. Thus, further studies on autoantibodies investigated in patients with OCD compared with healthy controls are needed.

## Introduction

Approximately 2% of the population worldwide suffer from obsessive-compulsive disorder (OCD; [1, 2]), frequently first presenting in childhood/adolescence or early adulthood [3]. The delayed diagnosis and disease burden results in considerable economic and psychological impairment [4, 5]. Patients with OCD tend to report poorer quality of life than, for example, patients with depression or even heroin addiction [6]. Core obsessive-compulsive symptoms (OCS) are ego-dystonic irrational obsessive thoughts that lead to time-consuming repetitive behaviors (compulsions) to reduce anxiety [7, 8]. Exposure therapy with response prevention as a form of cognitive behavioral therapy (CBT) constitutes the first choice in terms of psychotherapeutic approaches for OCD and serotonin reuptake inhibitors (SSRIs) are mainly used for psychopharmacotherapy [9–12]. Nevertheless, treatment resistance rates are high with approximately half of patients not responding sufficiently to first-line therapy [8].

There is increasing evidence that biological components have substantial influence on the development of this disorder. A moderate genetic component with heritability estimates ranging from 27 to 65% has been reported [13, 14]. Besides, epigenetic alterations were identified [15–17]. Neuroimaging studies point to an aberration of neuronal pathways involving cortico-striato-thalamo-cortical circuits [18, 19]. Electroencephalography (EEG) data suggest abnormalities in frontal areas of the brain and overstimulation regarding event-related potentials [20]. Furthermore, neurochemical investigations indicate an imbalance, especially in serotonergic, but also dopaminergic and glutamatergic neurotransmission [7, 8, 18, 21].

An autoimmune hypothesis in some cases was initially postulated based on an association between OCS in children and their exacerbation after infections with beta-hemolytic streptococci [22]. This subgroup of patients has been termed “pediatric autoimmune neuropsychiatric disorder associated with streptococcal infection”, or PANDAS. The pathogenic antibodies associated with the M-protein of beta-hemolytic streptococci are hypothesized to be able to cross the blood–brain barrier and cross-react with basal ganglia tissue, which may result in OCS [22–24]. Other studies corroborated this assumption by identifying anti-basal ganglia autoantibodies (ABGA) in children with PANDAS [25–28]. In line with this, Pearlman and colleagues [29] conducted a meta-analysis on ABGA in OCD patients and detected significantly elevated ABGA levels. However, several studies also suggest that other autoantibodies besides ABGA may be associated with OCD and OCS [30–35]. To date, however, systematic analyses on different autoantibodies in OCD are missing. Therefore, the aim of this systematic review is to summarize and analyze the findings on different autoantibodies in patients with OCD/OCS.

## Material and Methods

### Eligibility criteria

Original research findings comprising case reports, case series, uncontrolled and controlled cross-sectional studies of patients with OCD or OCS reporting on autoantibodies detected in blood/and or cerebrospinal fluid (CSF) were included. Patients of all ages were analyzed, and no restrictions were placed on the methodology of autoantibody testing.

Preliminary results as well as case reports/series and cross-sectional studies reporting findings of patients with OCD and comorbid Tourette syndrome were excluded. Studies conducted exclusively in animals or with pathogen-associated antibodies (e.g., streptococcal antibodies) were ruled out. Furthermore, all articles that were published in languages other than English or German were excluded.

### Outcome

The aim was to provide a descriptive presentation of autoantibody-associated case reports of patients with OCD, along with the findings from all cross-sectional studies.

### Literature research

The literature search was performed in PubMed in line with the PRISMA guidelines using the following search terms: “(OCD OR obsessive-compulsive OR obsessive OR compulsive) AND (antib* OR autoantib* OR auto-antib* OR immunoglob* OR IgG OR IgM OR IgA)”. All articles available until 17 February 2021 were searched. Titles and abstracts of all articles were screened independently by two expert raters (DD and KR). Subsequently, a full text analysis was conducted for papers that met the eligibility criteria. Disagreements were resolved by a third reviewer or consensus-based discussion. Additionally, references of literature reviews or meta-analyses specific to the immunological topic of the current literature search were screened for additional eligible references. In total, 13 reviews [30, 31, 33–43], and one meta-analysis [29] on autoimmunity in OCD were screened. In addition, the references of the included papers were screened. All publications could be accessed and none of the authors had to be asked specifically for an article.

## Results

### Search results

**Fig. 1:**
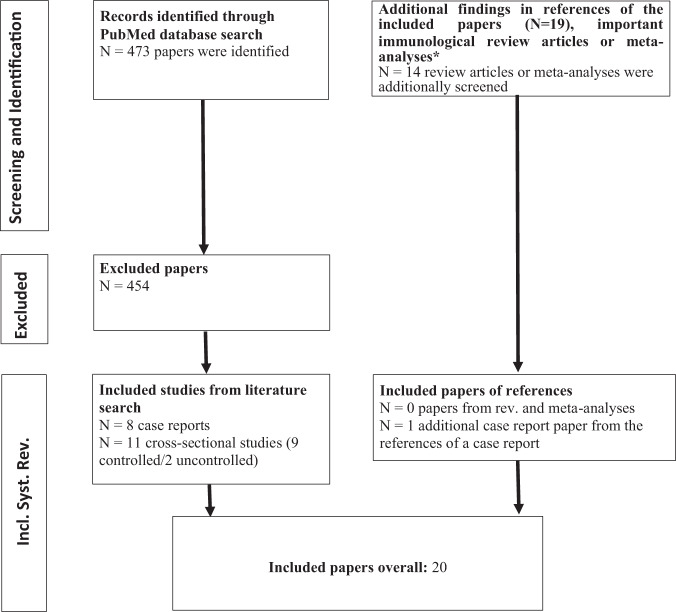
PRISMA flow diagram for systematic literature search. *Screened reviews and meta-analyses: [29–31, 33–43]. Additional case study from references of case report [45]: [49]. Included controlled studies into systematic review: [53, 55–61, 63]. Included uncontrolled studies into systematic review: [54, 62]. Included case reports into systematic review: [44–50, 52]. Incl. Inclusion, Syst. Systematic, Rev. Review.

The literature search resulted in a total of 473 reports. After screening, nine case reports [44–52] and eleven cross-sectional studies [53–63] were included. Of those, six studies included a healthy control group [53, 55–57, 61, 63], three included patient control groups [58–60], and two studies had no control group at all [54, 62]. The search results are summarized in Fig. 1.

### Systematic analysis of case reports

In the identified nine case reports, three patients with well-characterized anti-neuronal autoantibodies (against N-methyl-D-aspartate-receptor [NMDA-R], collapsin response mediator protein [CV2], paraneoplastic antigen Ma2 [Ma2]), two patients with non-well-characterized anti-neuronal autoantibodies (against voltage-gated potassium channels [VGKC], “anti-brain” structures), and four with autoantibodies associated with systemic autoimmune diseases (two with Sjögren syndrome, one with neuropsychiatric lupus, and one with anti-phospholipid autoantibodies) were described. The detailed findings are summarized in Table 1.

**Table 1:** Case-reports - Autoantibodies associated with obsessive-compulsive disorders (OCD) or obsessive-compulsive symptoms (OCS).

Study		Antibody	Diagnosis	Sex, Age	Laboratory Methods	Other Examinations	Diagnostic Findings	Treatment
**Well-characterized anti-neuronal autoantibodies**								
1. Cainelli et al. 2019 [44]	Anti NMDA-R Ab		Anti-NMDAR encephalitis with initial apathy and emerging OCS (controlling)	F, 8 years	Ab identification in CSF	MMSPE and neuro- psychological evaluation	CSF: Anti-NMDAR Ab with blood brain barrier dysfunction and no bacterial or neurotropic viruses Neuropsychological and memory tests: Impaired performance	Plasmapheresis and IV methylprednisolone → improvement in neuropsychological tests (residual symptoms remaining, OCS remaining)
2. Muehlschlegel et al. 2005 [48]	Anti-CV2 Ab		Paraneoplastic encephalitis with initial OCS (order)	F, 69 years	Ab identification in serum	Physical examination MRI Chest x-ray Brain biopsy Autopsy	Serum: Anti-CV2 Ab MRI: White matter lesions in striatum and globus pallidus, mild atrophy Physical examination: Progressing of severe neurological deficits Chest x-ray: Normal Brain biopsy: Cerebral amyloid; perivascular infiltration of T lymphocytes, non-specific gliosis Autopsy: “Dusky coloration” of basal ganglia; reduced caudate nucleus; extensive neuronal loss and perivascular and parenchymal infiltration of T-lymphocytes limited to the striatum, cerebral amyloid	Steroid and cyclophosphamide → no improvement, patient died 5 weeks later
3. Scheid et al. 2003 [50]	Anti-Ma2 Ab		Paraneoplastic encephalitis with initial OCD (checking)	M, 39 years	Ab identification in CSF and serum	Physical examination MRI EEG CSF Thoracic CT	Serum: Anti-Ma2 Ab CSF: Anti-Ma2 Ab EEG: Inconstant left temporal sharp slow-wave activity MRI: T2-hyperintense signal change in the left hippocampus Physical examination: Progressing of severe neurological deficits Histologic examination: Differentiated teratoma and seminoma-in-situ Thoracic CT: Metastasis of teratoma	IV immunoglobulins and lamotrigine → improvement of memory function and OCS
**Non-well characterized anti-neuronal autoantibodies**								
4. Celliers et al. 2016 [46]		Anti-VGKC Ab	VGKC-Ab associated limbic encephalitis with OCS and faciobrachial seizures	M, 64 years	Ab identification in CSF	FDG-PET EEG	CSF: Increased VGKC Ab FDG-PET: Increased symmetrical uptake in the caudate and lentiform nuclei EEG: No associated EEG-changes with seizures detected	Levetiracetam + IV methylprednisolone + mycophenolate mofetil → full remission of seizures and OCS; CSF: inconspicuous levels of VGKC Ab; FDG-PET: follow up showed significant glucose uptake reduction in the basal ganglia bilaterally
5. Zhu et al. 2014 [52]		IgG “ANAb”	Antibody associated OCS (checking, washing) after pineal germinoma	M, 23 years	Ab identification in serum	Physical examination MRI Brain biopsy	Serum: 43-45kDA, positive staining with Hep2 epithelial cells Physical examination: Severe neurological symptoms MRI: Enlarged pineal gland Brain biopsy: Germinoma	Cranial irradiation → OCS and Ab detection in serum (3 month later)
**Autoantibodies associated with systemic autoimmune diseases**								
6. Lüngen et al. 2019 [47]		ANA with anti-nucleosome specificity	OCS (with psychotic features) in the context of neuropsychiatric lupus	M, 22 years	Ab identification in CSF and serum	Physical examination EEG MRI FDG-PET	CSF: ANA pos., pleocytosis, OCBs Serum: ANA pos., complement factor C4 reduced and C3d increased Physical examination: Normal EEG: Slowing MRI: Multiple bilateral white matter lesions FDG-PET: Normal	IV methylprednisolone + MTX + HCQ → nearly full remission
7. Carvalho et al., 2020 [45]		ANA, anti-Ro/SS-A, anti-La/SS-B Ab	OCS (contamination, checking, washing) in the context of Sjögren syndrome	F, 40 years	Ab identification in serum	Lisamin green staining Break up time Schirmer test Scintigraphy Ultrasound	Serum: ANA pos., anti-Ro/SS-A, anti-La/SS-B Ab pos. Lisamin green staining: Pos. Schirmer test: Pos., break up time: Pos. Scintigraphy: Dysfunction of salivary glands Ultrasound: Gland dysfunction	HCQ + vitamin D3 + omega-3 → Improvement (OCS under adequate control)
8. Ong et al. 2017 [49]		ANA, anti-Ro/SS-A, anti-La/SS-B Ab	OCS (contamination, hoarding, symmetry, washing, cleaning, checking) with depression in the context of Sjögren syndrome	F, 17 years	Ab identification in serum	Physical examination Electrophoresis CSF Cerebral single photon emission CT MRI MRS	Serum: ANA pos., anti-Ro/SS-A, anti-La/SS-B Ab pos, RF Electrophoresis: Increase in polyclonal gammaglobulins Physical examination: “Fleeting sensation in the upper body”, tinnitus, rare visual hallucinations CSF: Pleocytosis, elevated protein, neopterin positive, CSF IgG elevation, significant increase of CSF IgG to albumin ratio, OCBs, no Ab MRI and cerebral single photon emission CT: Insignificant finding MRS: Reduction in N-acetylaspartate in both hippocampi	Plasmapheresis and IV immunoglobulins → complete remission of OCS, partial remission of depression
9. Sokol et al. 2006 [51]		Anti-phosphatidyl-ethanol-amine Ab	OCS (sudden onset after an ear infection)	F, 5 years	Ab identification in serum	Physical and neurological examination	Physical and neurological examination: Normal Blood: Repeated positive anti-phosphatidylethanol-amine levels, these correlated with the clinical course of OCD; no evidence of streptococcal infection	Improvement with low dose sertraline

Case reports that did not report OCD and Tourette syndrome separately were excluded as well as case studies reporting PANDAS/PANS. Patients from case series were included. *M* Male, *F* Female, *OCD* Obsessive-compulsive disorder, *PANDAS* Pediatric autoimmune neuropsychiatric disorders associated with streptococcal infections, *PANS* Pediatric acute-onset neuropsychiatric syndrome, *RF* Rheumatoid factor, *Ab* Autoantibody, *ANA* Antinuclear antibody, *ASO* Serum anti-streptolysin O, *CV2* Collapsin response mediator protein, *VGKC Ab* voltage gated potassium channel antibodies, *CSF* Cerebrospinal fluid, *MRI* Magnetic resonance imaging, *CT* Computed tomography, *MRS* Magnetic resonance spectroscopy, *OCBs* Oligoclonal bands, *OCS* Obsessive-compulsive symptoms, *MMSPE* Mini-Mental State Pediatric Examination, *BVN* Batteria di Valutazione Neuropsicologica per l’Età Evolutiva, *IV* Intra-venous, *IF* Immunofluorescence, *IgG* Immunoglobulin G, *kDa* Kilodaltons, *FDG-PET* 18F-Fluorodeoxyglucose positron-emission tomography, *MTX* Methotrexate, *HCQ* Hydroxychloroquine, *N.A.* (data) not available.

#### Specific autoantibodies against central nervous structures

##### NMDA-R

Seven patients in a case series with anti-NMDA-R encephalitis developed initial cognitive deficits and persisting impairment of neuropsychological function (mean follow-up = 35 months). One out of these seven patients developed persistent OCS [44].

##### CV2

OCS where the patient arranges and frequently rearranges objects in distinct lines, were the first symptoms diagnosed in a 69-year-old woman. Four weeks later, she showed initial neurological symptoms (choreatiform movements and unsteady gait, later followed by a loss of higher cortical functions) that steadily deteriorated until her death. Immunological treatment was unsuccessful. Brain autopsy revealed global cerebral amyloid with extensive neuronal loss and perivascular/parenchymal infiltration of T-lymphocytes limited to the striatum. Anti-CV2 autoantibodies were positive in the serum after her death. A tumor was not found, however, the autopsy was limited to the brain only [48].

##### Ma2

OCS consisting of checking central heating, light, doors, and so forth were the first signs of paraneoplasia in a 39-year-old male who later on started to develop severe neurological symptoms (memory decline, gustatory sensory auras, headache, etc.). Orchiectomy revealed a differentiated teratoma and seminoma in situ. Thoracic computer tomography (CT) confirmed metastasis of the teratoma. Magnetic resonance imaging (MRI) of the brain showed a T2 hyperintense signal alteration in the left hippocampus. EEG indicated left temporal sharp slow-wave activity, and laboratory analysis (CSF and serum) identified anti-Ma2 autoantibodies. Neither surgical removal of the pulmonary metastasis nor chemotherapy (bleomycin/cisplatin/etoposide) yielded clinical improvements in contrast to the antiepileptic and immunological treatment [50].

##### VGKC

A 64-year-old man without comorbidities presented with sudden OCS related to his garden (compulsively trimming of the lawn, especially the edges, with an extraordinary time investment). He additionally developed faciobrachial dystonic seizures. Anti-VGKC autoantibodies were significantly elevated in the CSF, whereas the other parameters were normal. Metabolic brain imaging with 18F-Fluorodeoxyglucose positron-emission tomography (FDG-PET) revealed increased symmetrical uptake in the caudate and lentiform nuclei while no association between seizures and EEG alterations were detected. After treatment with cytostatics and prednisolone, complete remission of OCS and seizure symptoms was observed, along with normalization of anti-VGKC autoantibody levels and reduced FDG uptake in the basal ganglia [46].

##### “Anti-brain”

Zhu and colleagues [52] described a case of a 23-year-old man who suffered from OCS three months after the irradiation of a pineal gland germinoma. “Anti-brain” autoantibodies were detected after symptom onset. These resembled antinuclear antibodies (ANAs) and showed a fine punctate pattern.

#### Autoantibodies associated with systemic autoimmune diseases

##### Neuropsychiatric lupus

OCS with psychotic tendencies were observed in a 22-year-old male patient who had ANAs with anti-nucleosome specificity detected in his blood and CSF accompanied by abnormalities in the MRI (multiple inflammatory bilateral white matter lesions) and EEG (slowing); improvement was reported by methylprednisolone, methotrexate, and hydroxychloroquine [47].

##### Sjögren syndrome

A 40-year-old female patient with xerostomia and xerophthalmia presented with OCS that included washing, checking behavior, and fear of contamination. The patient was diagnosed with OCD, and treatment with CBT and psychotropic drugs (fluoxetine/risperidone/aripiprazole) was initiated. Furthermore, the diagnosis of Sjögren syndrome was ascertained based on increased titers of ANAs with anti-Ro/SS-A and -La/SS-B specificity, a positive Schirmer test, and positive lisamin green staining. Ultrasound and scintigraphy showed dysfunction of the salivary glands. Both her rheumatological symptoms and OCS improved significantly after treatment with hydroxychloroquine and nutrition supplements [45].

Ong and colleagues (2017) described the case of a 17-year-old woman who initially presented with symptoms of OCD that included contamination fears, hoarding, symmetry, washing, cleaning and checking, and comorbid depression, which was managed with a low dosage of psychotropic drugs (fluoxetine, quetiapine, and prazosin) and psychotherapy. Subsequently, she developed severe neuropsychiatric symptoms (unusual sensations, visual hallucination, tinnitus, etc.) and was additionally diagnosed with Sjögren syndrome based on the positive titers of ANAs with anti-Ro/SS-A and anti-La/SS-B specificity. Magnetic resonance spectroscopy revealed a reduction in N-acetylaspartate in both hippocampi, while brain MRI presented insignificant findings. The CSF displayed pleocytosis, elevated protein, increased neopterin, immunoglobulin G (IgG) elevation, and a significant increase of CSF IgG to albumin ratio and oligoclonal bands, while anti-neuronal autoantibodies were negative. OCS completely disappeared with immunotherapy using plasmapheresis and intravenous methylprednisolone [49].

##### “Anti-phospholipid”

A 5-year-old girl developed rapid-onset OCD after an ear infection without evidence of streptococcal infection, but the patient had elevated anti-phosphatidylethanol-amine autoantibodies and the antibody levels correlated with OCS. Immunotherapy was not administered since she improved with low dose sertraline [51].

### Systematic review of cross-sectional studies

The findings are summarized in Table 2.

**Table 2:** Cross-sectional studies - Autoantibody findings received through the systematic literature search.

Study	Diagnoses	Autoantibody-Type	N (OCD Patients vs. Controls)	Sex	Age of Patients and Controls	Control Group	Methods	Statistics	Significance OCD vs. Controls	Special Features
**Anti-CNS autoantibodies**										
1. Yuwiler et al. 1992 [63]	OCD TS Schizophrenia MS ASS	Ab against serotonin	P: 6 C: 12	n.a.	P: 10 C: 33	HC	SS	Regression analysis	**Serum: S**	Significantly more binding inhibition in the sera of patients with OCD compared to HC.
2. Chain et al. 2020 [55]	OCD TS ADHD PANDAS SC	Tu, D1R, D2R, GM1, ANA, Anti-DNAase B, ASO	P: 25 C: 28	n.a.	n.a	HC	ELISA microtiter plate	Mann-Whitney U test, Wilcoxon signed-rank test, Fisher´s exact test	**Serum: S**	OCD-sera exhibited elevated Ab against D1R compared to HC-sera. No significant elevation for other Ab in OCD-Sera compared to HC.
3. Chiaie et al. 2012 [56]	OCD BD Schizophrenia PCD	PCA, ANA, AMA, ASMA, APCA	P: 7 C: 52	P: 1 F: 6M C: 26 F: 26 M	P: 31.72 ± 12.01 C: A = 43 5 ± 15.3	HC	IF	ANOVA / MANOVA	**Serum: S**	PCA were more prevalent in sera of OCD patients in comparison to HC.
4. Black et al. 1998 [54]	OCD	PCA, VGCC, AChR Bi, striated muscle Ab, Amph, ANA, SMA, AMA, Tg, Tp, GAD-65	P: 13	P: 6 F: 7 M	P: 39.92 ± 4.13	No controls	IP EI IF RAI MPA	No statistical approach	Serum: NS	The only 2 subjects with comorbid cancer (breast adenocarcinoma / thyroid carcinoma) exhibited elevated titer for microsomal autoantibodies.
5. Roy et al. 1994 [61]	OCD Schizophrenia AD MS HIV	Ab against somatostatin and dynorphin	P: 10 C: 25	P: 4 F: 6 M C: 15 F: 10 M	P: 31 ± 6 C: 40 ± 17	HC	ELISA	ANCOVA	**Serum: S**	Auto-Ab (somatostatin and dynorphin) were significantly elevated in the sera of patients with OCD in comparison to HC.
6. Singer et al. 2005 [62]	OCD OCD + ADHD TS-only TS + ADHD TS + ADHD + OCD ADHD	ABGA and other non-specific ANAb	P: 5	n.a.	n.a.	No controls	IHC ELISA	ANOVA and post hoc-t-test	Serum: NS	No comparison between the groups. No correlation between stereotypy scores of rats correlations toward infused ANAb titers.
7. Kirvan et al. 2006 [58]	OCD Tics ADHD PANDAS SC	ABGA (CSF) and GM1 / GlcNAc (serum)	P: 5 Tics: 10 ADHD: 10 PANDAS: 16 SC: 6	n.a.	n.a.	Psychiatric and neurological patients	IHC ELISA	ANOVA	Serum: NS CSF: NS	Low prevalence of lysoganglioside GM1 and GlcNAc in OCD-sera. No reactivity for human-caudate tissue in the CFS.
8. Nicholson et al. 2012 [60]	OCD Depression Schizophrenia	ABGA and ASO	P: 96 C: 50	P: 52 F: 44M C: 32 F: 18 M	P: 42.4 ± 13.3 C: 45.7 ± 13.1	Psychiatric patients (schizophrenia and depression)	WB	Fisher´s exact test	**Serum: S**	ABGA were significantly elevated compared to the heterogeneous psychiatric group. No significant correlation with ABGA-positivity in OCD and symptoms or clinical variables.
9. Gause et al. 2009 [57]	OCD OCD+Tics OCD + PANDAS	ABGA and Ab against cingulate gyrus, dorsolateral prefrontal and orbitofrontal cortex, ASO	P: 13 C: 29	P: 5 F: 8 M C: 17 F: 12 M	P: 14.1 ± 3.1 C: 12.4 ± 2.4	HC	WB IHC ELISA	ANOVA, Tukey´s post-hoc- test, chi-square test, Fisher´s exact test	Serum: NS	ASO titers did not correlate with immunoassays and were similar among the groups.
10. Bhattacharyya et al. 2009 [53]	OCD	ABGA and Ab against Thalamus	P: 23 C: 23	P: 5 F: 18 M C: 5 F: 18 M	P: 24.65 ± 8.95 C: 32 ± 12.95	HC	WB	Chi-square test/ MANCOVA	Serum: NS **CSF: S**	CFS glutamate and glycine levels were significantly higher in patients.
**Autoantibodies associated with systemic autoimmune diseases**										
11. Morer et al. 2006 [59]	OCD (early and late onset) psychiatric patients	ANA, AMA, APCA, ASMA, LKM, Tp, Tg, ASO	P (early onset): 18 P (late onset): 22 C: 14	P (early o.): 11 F: 7M P (late o.): 9 F: 13M C: 6 F: 8 M	P (early onset): 29.56 ± 8.29 P (late onset): 33.33 ± 9.04 C: 34.90 ± 10.60	Psychiatric patients (affective, adaptive, psychotic or anxiety disorders)	IF ELISA	Mann-Whitney U test, Wilcoxon signed-rank test, Fisher´s exact test	Serum: NS	No significant differences for autoimmune parameters in OCD-sera compared to the patient control group.

Studies that did not report OCD and Tourette syndrome separately were excluded as well as studies reporting PANDAS/PANS. Only studies reporting autoantibody findings are presented. All autoantibodies were illustrated in the table. Findings with significant group differences are marked in bold. *CNS* Central nervous system, *OCD* Obsessive-compulsive disorder, *TS* Tourette Syndrome, *ADHD* Attention deficit hyperactivity disorder, *AD* Alzheimer´s disease, *ASS* Autism spectrum disorder, *MS* Multiple sclerosis, *HIV* Patients with advanced immunodeficiency virus, *PANDAS* Pediatric autoimmune neuropsychiatric disorders associated with streptococcal infections, *PANS* Pediatric acute-onset neuropsychiatric syndrome, *BD* Bipolar disorder, *PCD* Paraneoplastic cerebellar degeneration, *P* Patient (only patients with OCD), *C* Control group, *HC* Healthy controls, *Ab* Autoantibody, *ABGA* Anti-basal ganglia autoantibodies, *ANAb* Anti-neuronal autoantibodies b, *Amph* Amphiphysin, *ANA* Antinuclear antibody, *AMA* Anti-mitochondrial autoantibody, *APCA* Anti-gastric parietal cell autoantibody, *ASMA* Anti-smooth muscle autoantibody, *ASO* Serum antistreptolysin O, *ELISA* Enzyme-linked Immunosorbent Assay, *AChR* Acetylcholine receptor-binding autoantibodies, *D1R/D2R* Dopamine receptor autoantibodies, *GM1* Lysoganglioside GM1 autoantibody, *LKM* Anti-liver-kidney microsome autoantibody, *PCA* Purkinje cell cytoplasmic autoantibodies, *SMA* Smooth muscle autoantibody, *GAD-65* Glutamic acid decarboxylase-65-kDa isoform autoantibody, *Tg* Thyroglobulin autoantibody, *Tu* Tubulin autoantibody, *Tp* Thyroid microsomal (peroxidase) autoantibody, *VGCC* N-type and P/Q-type voltage gated calcium channel autoantibodies, *N* Number, *F* Female, *M* Male, *A* Mean age, *WB* Western blotting, *IF* Immunofluorescence, *IHC* Immunohistochemistry, *IP* Immunoprecipitation assays, *EI* Enzyme immunoassay (not explained in more detail whether ELISA or other test), *RIA* Radioimmunoassay, *MPA* Microtiter particle agglutination test, *SS* Scintillation spectrometry, *CSF* Cerebrospinal fluid, *S* Significant, *NS* Not significant, *N.A.* (data) not available, *o.* Onset.

#### Specific autoantibodies against central nervous structures

In a sample of mixed mental disorders (*N* = 48) comprising seven patients with OCD and 52 healthy controls, two out of seven patients with OCD were positive for autoantibodies against Purkinje cells, for which all healthy volunteers were negative. Overall, Purkinje cell antibodies were identified in 11 of 48 patients (22.9%) with mental disorders but in 0% of controls [56]. Another study showed that a significant proportion of patients with OCD (*N* = 25) possessed IgG serum autoantibodies against the dopamine D1 receptor, while other anti-CNS autoantibody findings (dopamine D2 receptor, lysoganglioside GM1, and tubulin) were detected in comparable rates in healthy controls (*N* = 28) [55]. Ten patients with OCD exhibited significantly elevated serum autoantibodies to somatostatin and prodynorphin compared with 25 healthy controls and patients with advanced immunodeficiency virus (HIV), schizophrenia, Alzheimer’s disease, and multiple sclerosis [61]. Singer et al. [62] measured the titers of anti-neuronal autoantibodies b [“ANAb”] in the sera of patients with mental disorders (including five patients with OCD) using different methods. Two out of five patients with OCD showed low titers for ANAb [62]. Serum IgG autoantibodies reacted with lysoganglioside GM1 and GlcNAc (epitope of streptococcus) in only one out of five OCD-sera (20%), while it reacted in all the Sydenham’s chorea-sera (100%), 11 out of 16 PANDAS-sera (69%), 3 out of 10 Tic-sera (30%), and 2 out of 10 ADHD-sera (20%). OCD and other non-PANDAS CSF failed to show reactivity for human caudate-putamen tissue compared with patients with PANDAS [58]. In a larger cohort of 96 patients with OCD, ABGA was significantly elevated in 19 out of 96 participants compared with a heterogeneous psychiatric group (*N* = 50, schizophrenia and depression, elevation in 2 out of 50). Most positive OCD-sera displayed anti-enolase autoantibodies (13 out of 19), as measured by immunoblotting [60]. Bhattacharyya and colleagues [53] included OCD-only patients and healthy controls when comparing ABGA concentration levels. They did not find significant differences of ABGA in OCD compared with healthy controls. However, Bhattacharyya et al. [53] showed that the binding of CSF autoantibodies to the thalamus and basal ganglia was significantly higher in patients (*N* = 23) than in controls (*N* = 23), whereas no significant difference was found when the autoantibodies were examined in sera. In addition, the concentration of CSF glutamate and glycine levels was higher in patients compared to controls [53].

One study analyzed the correlation between blood serotonin concentration in patients with OCD (*N* = 6) and healthy controls (*N* = 12) and the presence of IgG autoantibodies inhibiting serotonin binding to the human frontal cortex was positive but not significant. However, the serotonin concentration of patients with OCD was lower and binding inhibition significantly higher in comparison to healthy volunteers [63]. Another study that examined the serum of 13 patients with OCD did not find any specific pattern involving anti-neuronal autoantibodies (against Purkinje cells, N-type and P/Q-type voltage-gated calcium channels, neuronal nuclear epitopes, amphiphysin, and glutamic acid decarboxylase) or systemic autoantibodies [54]. Although two individuals had elevated titers for anti-microsomal autoantibodies. These were the only participants in this cohort with a cancer diagnosis (breast adenocarcinoma and thyroid carcinoma) [54]. Gause and colleagues [57] included OCD-only patients and healthy controls when comparing ABGA concentration levels. Gause and collegues did not find significant differences of ABGA in OCD compared with healthy controls. Three methods (ELISA, immunohistochemistry, Western blot) were utilized, but no significant group differences (13 OCD patients and 29 controls) were identified [57].

#### Autoantibodies associated with systemic autoimmune diseases

Serum autoantibodies such as ANAs, anti-mitochondrial autoantibodies [AMA], anti-gastric parietal cell autoantibodies [APCA], anti-smooth muscle autoantibodies [ASMA], as well as antibodies against liver-kidney microsome [LKM]/ thyroid microsomal (peroxidase) [Tp]/ thyroglobulin [Tg]/ and streptolysin O [ASO]) were screened in a study of 40 OCD patients and compared to 14 patients with other mental disorders including mood, adjustment, psychotic, or anxiety disorders. Furthermore, OCD patients were divided into two subgroups based on disease onset (early- vs. late-onset OCD). No significant differences in autoantibody prevalence emerged between OCD subgroups and controls. However, early-onset OCD was associated with higher anti-streptolysin O levels [59].

## Discussion

This systematic literature review examined the association between OCD/OCS and different autoantibodies. It includes all articles on this topic published until February 2021 to provide a comprehensive and broad overview. Patients with PANDAS and PANS patients were not content of the paper. Nine case reports of autoantibody-associated OCD have been published, of which six patients (67%) benefited from immunotherapy. Six of eleven cross-sectional studies have also provided evidence for an association between autoantibodies and OCD.

### Anti-CNS autoantibodies from serum and CSF

A total of five patients with OCS described as case reports had anti-CNS autoantibodies [44, 46, 48, 50, 52]; out of those, three patients had well-characterized anti-neuronal autoantibodies (against NMDA-R, CV2 and Ma2), and two patients had autoantibodies against non-well-characterized neuronal antigens (VGKC and “anti-brain”). The patient with anti-NMDA-R autoantibodies was autoantibody positive in CSF and responded well to immunotherapy [44], suggesting a causal role of these autoantibodies in this patient [64]. In line with this, Al-Diwani et al. [65] showed that 2% of all patients with anti-NMDA-R encephalitis exhibited OCS. The two patients with paraneoplastic anti-neuronal autoantibodies against intracellular antigens (CV2 and Ma2; 48, 50) had severe courses. In both patients, OCS consisted of early symptoms of paraneoplastic encephalitis; this suggests that a paraneoplastic cause could be considered in patients with late onset OCS or additional tumor disease or neurologic symptoms. Nevertheless, these results should be interpreted with caution, as this observation is based on a small number of cases. Anti-Ma2 autoantibodies were also detected in the CSF, and typical autopsy findings with infiltration of T-lymphocytes to the striatum were found in the patient with anti-CV2 autoantibodies. Thus, it might be assumed that these three cases were not only associated with irrelevant serum autoantibodies [66–69]. Anti-VGKC autoantibodies are increasingly considered non-specific, and a determination of anti-leucine-rich, glioma inactivated 1 (anti-LGI1) and contactin-associated protein-2 (CASPR2) autoantibodies is suggested [70]. However, the OCD patient with anti-VGKC autoantibodies showed typical findings of limbic encephalitis with anti-VGKC autoantibody detection in the CSF and additional faciobrachial dystonic seizures [46, 70]. Thus, this patient might have had functional autoantibodies as well, which is also supported by the response to immunotherapy. Another patient showed novel “anti-brain” autoantibodies [52]. These “anti-brain” autoantibodies have not yet been investigated in larger cross-sectional studies (see Table 2). However, a small previous study identified a more frequent occurrence of autoantibodies against Purkinje cell targets [56]. Such immunofluorescence patterns are mostly found in patients with paraneoplastic anti-neuronal autoantibodies [71]. Future studies are necessary to investigate a possible association of OCD with well-characterized anti-neuronal autoantibodies against (paraneoplastic) intracellular or cell surface antigens.

Previous cross-sectional studies have mostly focused on autoantibodies associated with dysfunction along the cortico-striato-thalamo-cortical circuits and serotonin or dopamine receptor pathways, based on established OCD pathophysiology. In this context, several studies investigated ABGA [53, 57, 58, 60, 62], and one study investigated autoantibodies against serotonin [63] while another explored autoantibodies against dopamine receptors [55]. ABGA was first described in Sydenham’s chorea, a disease associated with streptococcal infection [72] and later for OCD-related disorders such as Tourette syndrome [73, 74] and PANDAS (25, 26; 27). ABGAs appear to be the most studied autoantibodies in trials in patients with OCD, and several studies indicate a link between ABGAs and OCD [53, 60]. A meta-analysis by Pearlman and colleagues [29], which included six studies and one meeting abstract, reported significant elevation of ABGA in patients with OCD compared with controls [29]. However, Pearlman and colleagues [29] included studies comparing OCD patients with heterogeneous groups of psychiatric or neurological controls including comorbid Tourette syndrome. Tourette syndrome may be linked with immunological processes and different autoantibodies itself [73, 75, 76], and, ABGA in patients with OCD and comorbid Tourette syndrome has been strongly correlated with Tourette syndrome [25]. A comparison of patients with OCD to patients with other mental or neurological disorders may be vulnerable because many studies showed immunological findings in these disorders as well [32, 77–80]. Bhattacharyya and colleagues [53] revealed no significant difference in the serum autoantibodies binding to basal ganglia homogenate, whereas CSF ABGA was significantly elevated in patients with OCD compared with healthy controls. The same was true for autoantibodies against the thalamus [53]. This might be related to intrathecal autoantibody synthesis. Further studies are warranted to analyze CSF autoantibodies in OCD and healthy controls to differentiate whether CSF autoantibodies are more specific biomarkers than serum autoantibodies. The studies on autoantibodies preventing serotonin binding as well as on the dopamine D1 receptor [55, 63] also yielded promising results. Nonetheless, each autoantibody has been investigated in only one study to date and should be replicated in future studies.

### Systemic autoantibodies from serum and CSF

Four case reports with autoimmune forms of OCD in the context of systemic autoantibodies were identified [45, 47, 49, 51]. Three patients suffered from connective tissue disorders (one patient with neuropsychiatric lupus [47], two patients with Sjögren syndrome [45, 49], and one patient was positive for anti-phospholipid autoantibodies [51]. All three patients with connective tissue disorders were identified based on their systematic autoantibodies. Two out of the three patients also showed inflammatory CSF changes suggesting neuroinflammation. In line with this, all three patients benefitted from immunotherapy. Thus, these three cases suggest that immunotherapies may possibly contribute to the reduction of OCS in the context of connective tissue disorders. In a smaller study with 40 patients, several systemic autoantibodies (ANA, AMA, APCA, as well as antibodies against LKM, Tp, Tg, and ASO) were investigated and no significant differences for these autoimmune parameters in OCD-sera compared to the patient control group were identified compared with patients suffering from other mental disorders including patients with psychotic and mood disorders [59]. However, this is not surprising, since an association with connective tissue disorders has also been reported in psychosis and mood disorders [81]. In two other studies focusing on anti-neuronal autoantibodies, ANAs were coinvestigated [54, 56], but here, only 20 patients with OCD were screened. Therefore, further research seems urgently necessary. Looking at the course of OCD, it is quite similar to the progression of connective tissue disorders such as systemic lupus erythematosus with an often insidious development of the disease in both entities [82]. The need for research about the link between OCD and connective tissue disorders is supported by the results of two recent cohort studies: A nationwide study from Taiwan suggested a clear association between OCD and systemic lupus erythematosus, Sjögren syndrome, and dermatomyositis [83]. Another study from Sweden reported an autoimmune concomitant disorder in 43% of patients with OCD [84].

### Limitations

Only PubMed (and the references of included papers and important reviews) were searched. Specific limitations related to the data from patients with OCD in the included studies were as follows: Several studies were lacking data for age and sex [55, 58, 62], investigated mostly small numbers of patients with OCD [54, 56–58, 61–63], had mixed control groups [59, 60], had unbalanced group ratios [56, 57, 61, 63] or did not include controls at all [54, 58–60, 62]. Overall, the studies with healthy control subjects showed more frequent abnormal findings, suggesting that future studies should use healthy control groups for reliable results. The current body of literature seems too narrow to ensure a reliable scientific conclusion on the prevalence and relationship of autoantibodies with OCD/OCS, or to provide reliable information on differences to other mental disorders such as psychosis or depression (for which more studies are available). Further controlled studies in large cohorts of multimodal investigated patients with the latest autoantibody testing methods (fixed cell-based assays, live cell-based assays, immunoblots, and/or tissue-based assays using indirect immunofluorescence) are needed.

## Conclusions

Case reports suggest a possible association of OCS with autoantibodies. Some cross-sectional studies also showed correlations between OCD and different autoantibodies in rare cases, but were mostly conducted with small sample size or were not replicated. Therefore, larger studies analyzing different autoantibodies are needed. The concept of a rare subtype of “autoimmune OCD” should be further investigated as it may open up novel avenues for targeted therapeutic approaches in the future.

## Data Availability

All relevant findings are presented descriptively in the paper.
